# *In Vitro* and *In Vivo* Studies on the Antibacterial Activity and Safety of a New Antimicrobial Peptide Dermaseptin-AC

**DOI:** 10.1128/Spectrum.01318-21

**Published:** 2021-12-15

**Authors:** Jiajia Chen, Doudou Hao, Kai Mei, Xin Li, Tingting Li, Chengbang Ma, Xinping Xi, Lei Li, Lei Wang, Mei Zhou, Tianbao Chen, Jia Liu, Qing Wu

**Affiliations:** a School of Chinese Materia Medica, Beijing University of Chinese Medicine, Beijing, China; b Natural Drug Discovery Group, School of Pharmacy, Queen’s University, Belfast, UK; Hartford Hospital

**Keywords:** bacterial resistance, antimicrobial peptides, Dermaseptin

## Abstract

Antimicrobial resistance has been an increasing public health threat in recent years. Antimicrobial peptides are considered as potential drugs against drug-resistant bacteria because they are mainly broad-spectrum and are unlikely to cause resistance. In this study, a novel peptide was obtained from the skin secretion of Agalychnis callidryas using the “shotgun” cloning method. The amino acid sequence, molecular weight, and secondary structure of Dermaseptin-AC were determined. The *in vitro* antimicrobial activity, hemolysis, and cytotoxicity of Dermaseptin-AC were evaluated. MICs and minimum bactericidal concentrations (MBCs) of Dermaseptin-AC against seven different bacterial strains ranged between 2 ∼ 4 μM and 2 ∼ 8 μM. The HC_50_ (50% maximum hemolysis concentration) of Dermaseptin-AC against horse erythrocytes was 76.55 μM. The *in vivo* anti-MRSA effect was tested on immune-suppressed MRSA pneumonia in mice. Dermaseptin-AC showed anti-MRSA effects similar to the same dose of vancomycin (10 mg/kg body weight). Short-term (7 days of intraperitoneal injection, 10 mg/kg body weight) *in vivo* safety evaluation of Dermaseptin-AC was tested on mice. The survival rate during the 7-day injection was 80%. Dermaseptin-AC showed no obvious effect on the liver, heart, spleen, kidney, and blood, but did induce slight pulmonary congestion. The skin safety of Dermaseptin-AC was evaluated on wounds on the back skin of a rat, and no irritation was observed.

**IMPORTANCE** In this study, we discovered a new antimicrobial peptide, Dermaseptin-AC, and studied its *in vitro* and *in vivo* antimicrobial activity. These studies provide some data for finding new antimicrobial peptides for overcoming antimicrobial resistance. Dermaseptin-AC showed strong broad-spectrum antibacterial activity and relatively low hemolysis, and was more cytotoxic to cancer cells than to normal cells. Dermaseptin-AC was active *in vivo*, and its anti-MRSA effect was similar to that of vancomycin when administered by intraperitoneal injection. Safety studies found that continuous injection of Dermaseptin-AC may cause mild pulmonary congestion, while there was no obvious irritation when it was applied to skin wounds. Chronic wounds are often accompanied by high bacterial burdens and, at the same time, antimicrobial resistance is more likely to occur during repeated infections and treatments. Therefore, developing Dermaseptin-AC to treat chronic wound infection may be an attractive choice.

## INTRODUCTION

Antimicrobial resistance (AMR) is a serious global problem that poses a burden on health and economy. For decades, antibacterial drugs have been widely overused and even accumulated along the food chain (1, 2). Therefore, the efficacy of antibacterial drugs has been reduced with the emergence of AMR (3). Hospitals on all continents of the world are currently plagued by AMR infections, and these microorganisms can even exist in a hospital for a long time (>8 years) (4). In particular, data showed that approximately 70% of hospitalized COVID-19 patients have received antibiotics, which may aggravate the overuse of antibiotics and accelerate the occurrence of AMR (5). If there is no new treatment, it is estimated that the death rate due to incurable infections will increase more than 10-fold by 2050 (6). In particular, chronic wounds are often associated with a high bacterial burden, which in turn promotes AMR (7). On the issue of AMR, methicillin-resistant Staphylococcus aureus (MRSA) undoubtedly has a major impact on public health, because it is a common cause of hospital and community-acquired infections (8).

Antimicrobial peptides (AMPs) could produce antibacterial effects by affecting bacteria cell shape and membrane permeability (9). The biochemical and pharmacodynamic properties of AMPs make them more resistant to AMR than conventional antibiotics (10). Thus, the diversity and effectiveness of AMPs make them attractive candidates for avoiding the current resistance crisis faced by conventional antibiotics. The complex envelope which surrounds microorganisms (including the inner and outer membranes) is the main obstacle for almost all antibacterial agents. AMPs can penetrate this barrier by forming micro micelles on the membrane, or by directly passing through the lipid bilayer (11). Moreover, the combined use of AMPs and classic antibiotics has the potential to help break through the bacterial cell barrier and enhance the transport of antibiotics (12). Amphiphilic α-helical cationic AMPs are found in many frog species, and it is believed that they have withstood the test of AMR in the course of long-term evolution (13). AMPs of the dermaseptin family have shown effectiveness against bacteria, parasites, fungi, protozoa, viruses, and several human cancer cells *in vitro*, as well as showing immune-modulatory effects (14).

In this study, a new AMP, Dermaseptin-AC, was discovered from the skin secretion of a red-eyed tree frog (*Agalychnis callidryas*) using the “shotgun” cloning method. Dermaseptin-AC showed strong broad-spectrum antibacterial activity and relatively slight hemolysis. *In vivo* experiments confirmed that Dermaseptin-AC effectively reduced MRSA pneumonia on immunosuppressed mice, and its efficacy was close to that of vancomycin. The short-term *in vivo* safety evaluation showed that Dermaseptin-AC did not affect the blood, liver, heart, spleen, or kidney of healthy mice, but it could induce mild pulmonary congestion. Dermaseptin-AC appeared to cause no irritation on skin wounds on a rat, so it has potential for treating drug-resistant infections in chronic wounds.

## RESULTS

### cDNA sequence, amino acid sequence, and peptide family.

**FIG 1:**
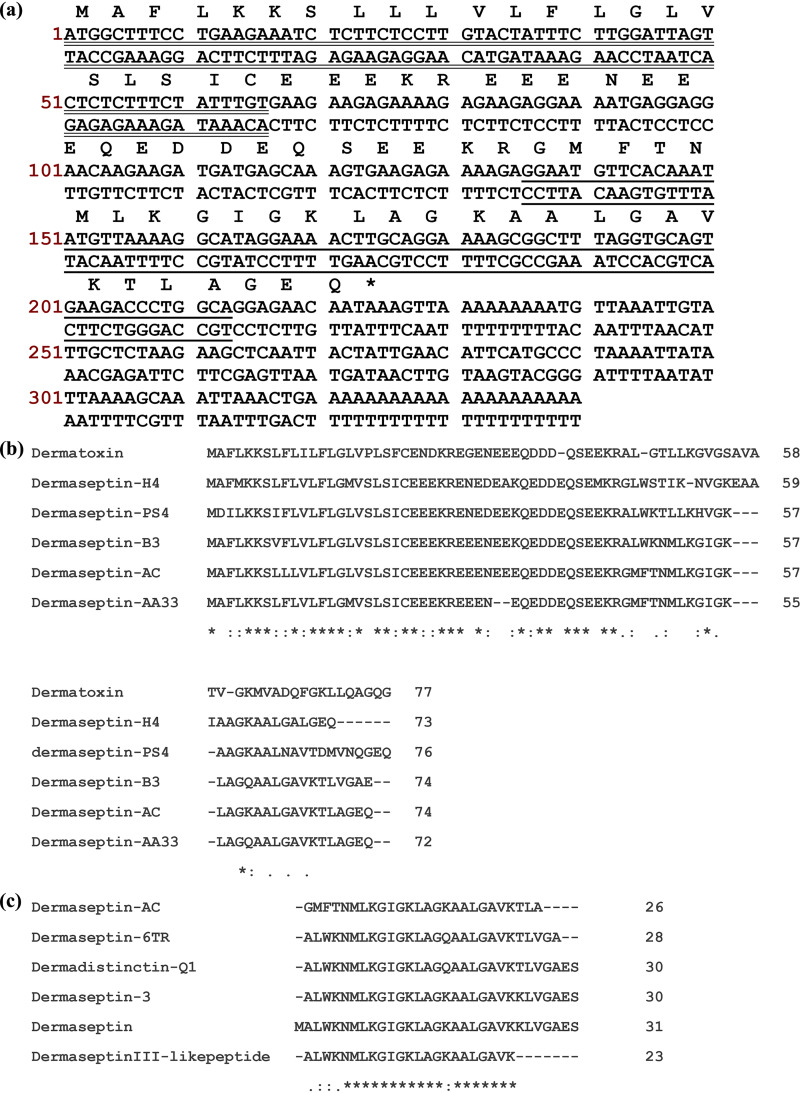
Discovery of Dermaseptin-AC. (a) Nucleotide sequence of the cDNA-encoding precursor of the novel peptide Dermaseptin-AC, and the amino acid sequence translated by the open reading frame. Signal peptide sequence is double underlined. Mature peptide sequence is underlined. (b) Amino acid sequence alignment of Dermaseptin-AC precursor and the top 5 similar peptide precursors. (c) Amino acid sequence alignment of the mature peptide of Dermaseptin-AC and the top 5 similar peptides. Symbols: * (asterisk), conserved residues; : (colon), very similar residues; . (period), similar residues.

The coding region of the peptide precursor consists of 74 amino acid residues, as shown in Fig. 1a. The signal peptide region starts from the N-terminus with methionine (M) and ends with cysteine (C), with a total length of 22 amino acid residues. This is followed by an acidic spacer peptide region of 23 amino acid residues, which ends with the convertase cleavage site -lysine-arginine- (-KR-). Finally, there is a mature peptide region of 26 amino acid residues with an untranslated region at the C-terminus. The mature peptide sequence was thus deduced as GMFTNMLKGIGKLAGKAALGAVKTLA-NH_2_. The sequences of five most similar peptides or peptide precursors were searched and aligned with the newly discovered peptide according to the BLAST analysis, as shown in Fig. 1b and c. From the BLAST results, the novel peptide was inferred to belong to the dermaseptin family and was thus named “Dermaseptin-AC.”

### Synthesis, purification, and secondary structure determination.

**FIG 2:**
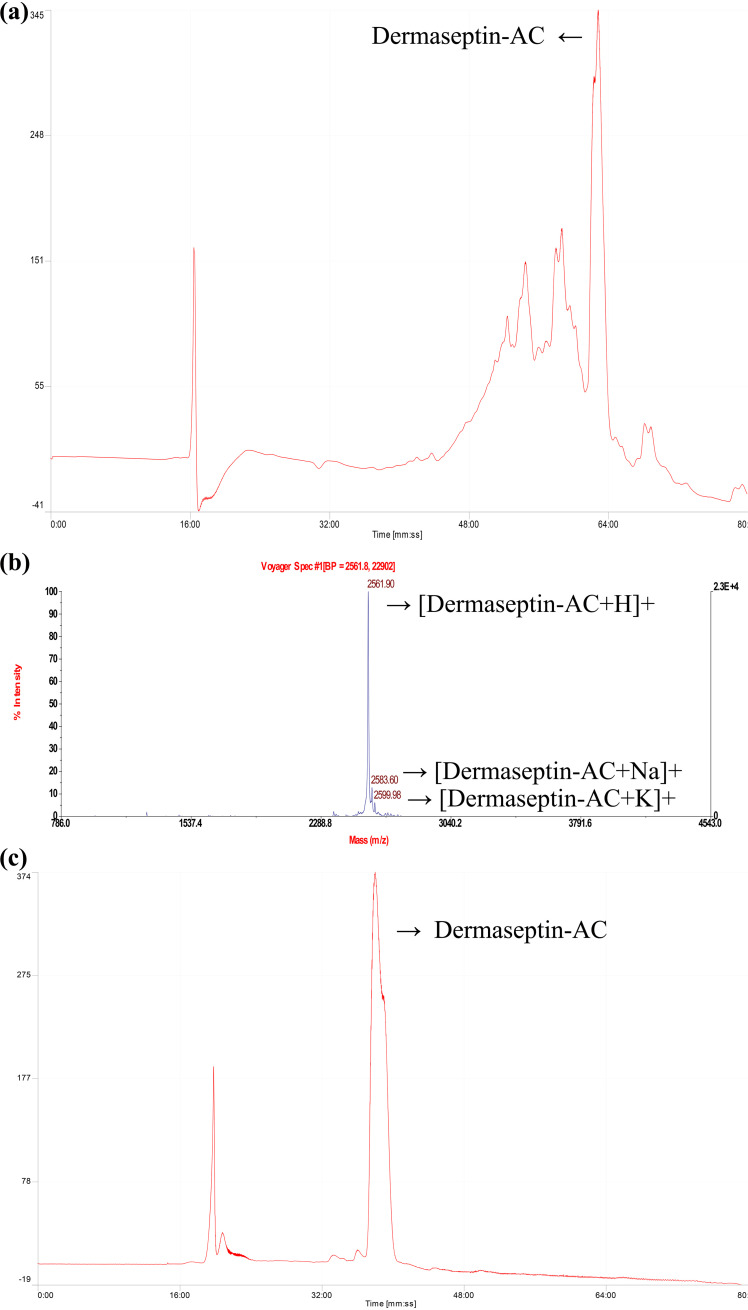
Synthesis and purification of Dermaseptin-AC. (a) HPLC chromatogram of synthetic peptide. The *y* axis indicates absorbance at λ = 214 nm. Linear gradient elution from 100% solution A (80% ACN, 19.95% water, 0.05% TFA) to 100% solution B (0.05% TFA, 99.95% water), 80 min, flow rate 1 mL/min. (b) MALDI-TOF spectrum of purified peptide. (c) HPLC chromatogram of purified peptide. The *y* axis indicates absorbance at λ = 214 nm. Linear gradient elution from 70% solution A (80% ACN, 19.95% water, 0.05% TFA) to 100% solution B (0.05% TFA, 99.95% water), 80 min, flow rate 1 mL/min.

**FIG 3:**
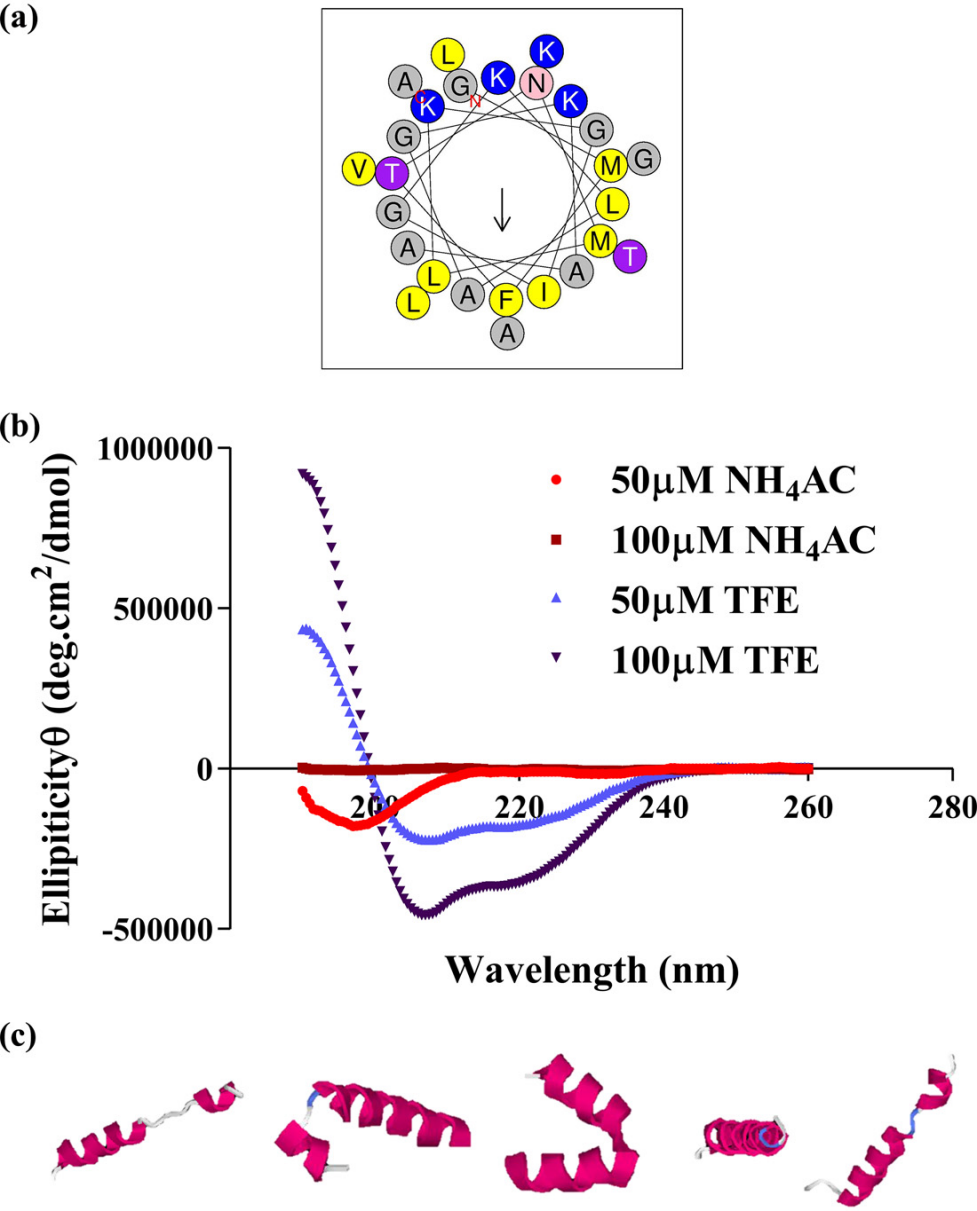
Structure of Dermaseptin-AC. (a) Amino acid composition of Dermaseptin-AC presented by HeliQuest. (b) Circular dichroism spectra recorded for the purified synthetic Dermaseptin‐AC in 50 μM NH_4_Ac, 100 μM NH_4_Ac, 50 μM TFE, and 100 μM TFE water solution. (c) 3D structure models of Dermaseptin-AC as predicted by I-TASSER.

In the high performance liquid chromatogram (HPLC), the peak with a retention time of 62.53 min was identified as Dermaseptin-AC, as shown in Fig. 2a. The purified peptide was analyzed by matrix-assisted laser desorption ionization–time of flight (MALDI-TOF) mass spectrometer, and the result showed that the impurity intensity of purified peptide was less than 5%, as shown in Fig. 2b. Molecular weight of the peak with highest intensity (%) was 2,561.90 (av.) Da, representing protonated Dermaseptin-AC. The peak with a molecular weight of 2,583.60 Da represents Dermaseptin-AC with the sodium ion. The peak with a molecular weight of 2,599.98 Da represents Dermaseptin-AC with the potassium ion. The HPLC chromatogram indicated that the purity of the peptide was no less than 95%, as shown in Fig. 2c. Physicochemical properties and amino acid composition of Dermaseptin-AC were calculated by the web server HeliQuest. The result showed that the hydrophobicity, hydrophobic moment, and net charge of Dermaseptin-AC were 0.445 H, 0.378 μH, and +5, respectively. The amino acid composition is shown in Fig. 3a. The secondary structure of pure Dermaseptin-AC was determined by circular dichroism spectra. Under physiological conditions, Dermaseptin-AC adopts an α-helix structure similar to that of typical cationic AMPs, as shown in Fig. 3b. The circular dichroism spectrum of Dermaseptin-AC in 50% TFE-10 mM NH_4_Ac solution (simulating the cell membrane) was analyzed by BeStSel, and showed a 50.2% alpha helix structure (15). The protein structure modeling software I-TASSER predicted the 3D structure of Dermaseptin-AC, as shown in Fig. 3c.

### *In vitro* antimicrobial activities.

**FIG 4:**
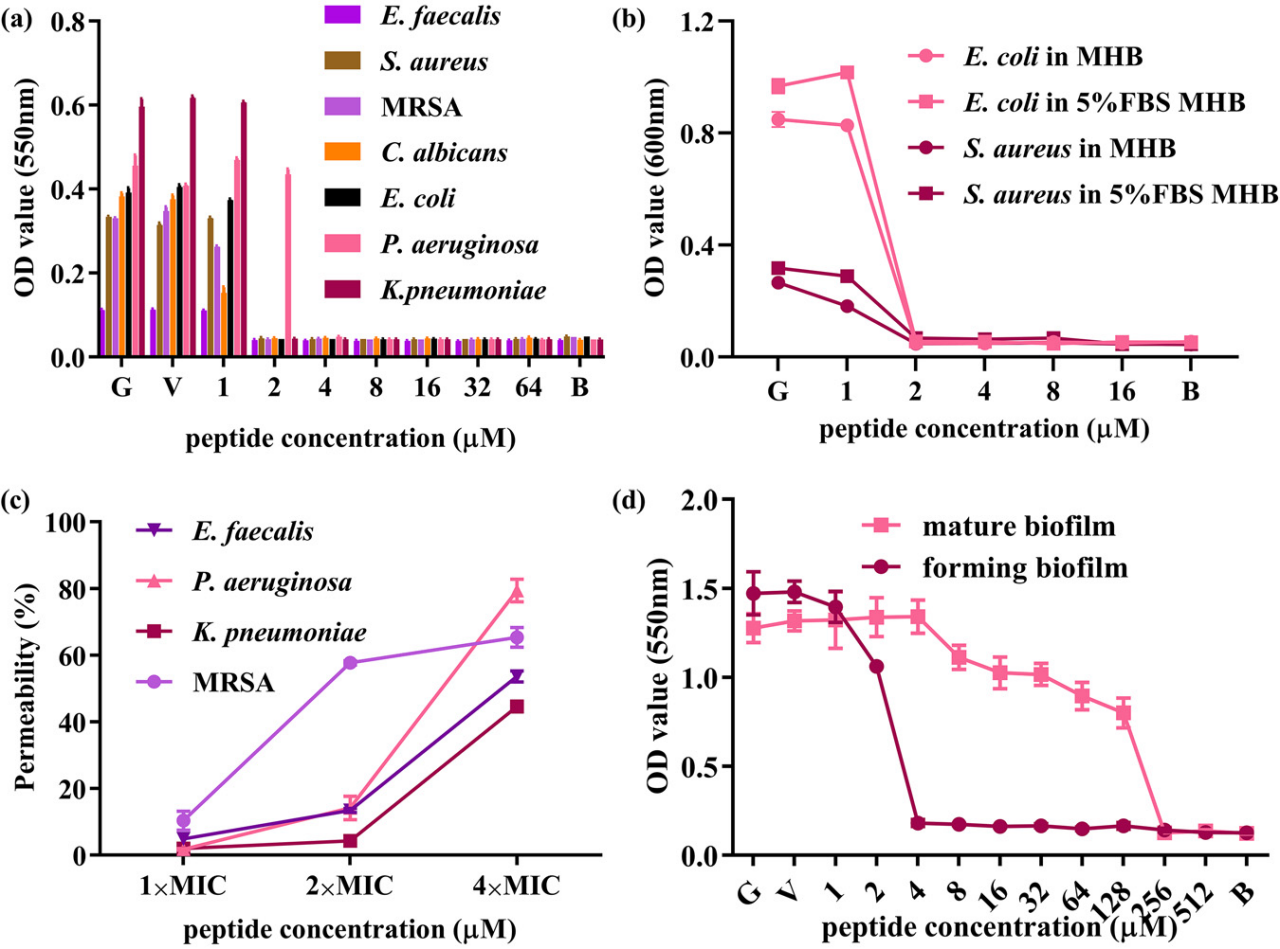
*In vitro* antimicrobial activity of Dermaseptin‐AC. (a) The *in vitro* antimicrobial activity of Dermaseptin‐AC. All strains were incubated with the peptide in Mueller-Hinton broth (MHB) at 37°C for 18 ∼ 20 h. (b) Effect of serum on the antimicrobial activity of Dermaseptin‐AC. Bacteria were incubated with the peptide in MHB or MHB with 5% fetal bovine serum at 37°C for 18 ∼ 20 h. (c) Membrane permeability of bacteria under Dermaseptin‐AC treatment. Bacteria were incubated with peptide or 70% isopropanol at 37°C for 2 h and then stained by SYTOX Green. Bacteria treated with 70% isopropanol were set as 100% permeabilized. Permeability was calculated from the measured fluorescence intensity. (d) Inhibition and eradication activities of Dermaseptin‐AC against MRSA biofilm. Forming biofilm was incubated with peptide in tryptic soy broth (TSB) at 37°C for 24 h. Mature biofilm was cultured in TSB at 37°C for 48 h and then incubated with peptide at 37°C for 24 h. Floating bacteria were rinsed off and the biofilm was stained with 0.1% crystal violet solution. B represents blank control (sterile medium), V represents vehicle control (1% DMSO), and G represents growth control. All data were analyzed by GraphPad Prism (Version 8.0.2), shown as mean ± SEM of 3 individual experiments with 3 replicates in each.

Dermaseptin-AC exhibited antimicrobial activities against all tested microorganisms, including S. aureus, E. faecalis, MRSA, E. coli, K. pneumoniae, P. aeruginosa and C. albicans. The MIC, MBC (minimum bactericidal concentration), and MBC/MIC values of Dermaseptin-AC against the above strains is summarized in Table 1. The antibacterial activity of Dermaseptin-AC between serum-containing and serum-free medium was compared, and results showed that the MIC and MBC values of Dermaseptin-AC against S. aureus and E. coli did not change. SYTOX Green is a green fluorescent nucleic acid stain that cannot penetrate intact cell membranes, so its fluorescence intensity indicates changes in cell membrane permeability. Results of SYTOX Green staining assay showed that Dermaseptin-AC increased the membrane permeability of E. faecalis, P. aeruginosa, K. pneumoniae and MRSA at 4×MIC. However, only the permeability of MRSA membrane was changed at 2×MIC. In addition, at the concentration of 4 μM, Dermaseptin-AC inhibited biofilm formation in MRSA. On mature MRSA biofilms, Dermaseptin-AC exhibited an eradicating effect at 256 μM. The above data are shown in Fig. 4.

**TABLE 1:** MIC and MBC values of Dermaseptin-AC in Mueller-Hinton broth and agar

Bacterial strain	Concn (μM, μg/mL)		MBC/MIC
	MIC	MBC	
Staphylococcus aureus (NCTC 10788)	2, 5.122	2, 5.122	1
Enterococcus faecalis (NCTC 12697)	2, 5.122	2, 5.122	1
MRSA^*a*^ (ATCC 43300)	2, 5.122	2, 5.122	1
Escherichia coli (NCTC 10418)	2, 5.122	2, 5.122	1
Klebsiella pneumoniae (ATCC 43816)	2, 5.122	8, 20.488	4
Pseudomonas aeruginosa (ATCC 27853)	4, 10.244	8, 20.488	2
Candida albicans (NCYC 1467)	2, 5.122	2, 5.122	1

a MRSA, Methicillin-resistant Staphylococcus aureus.

### Hemolysis and cytotoxicity.

**FIG 5:**
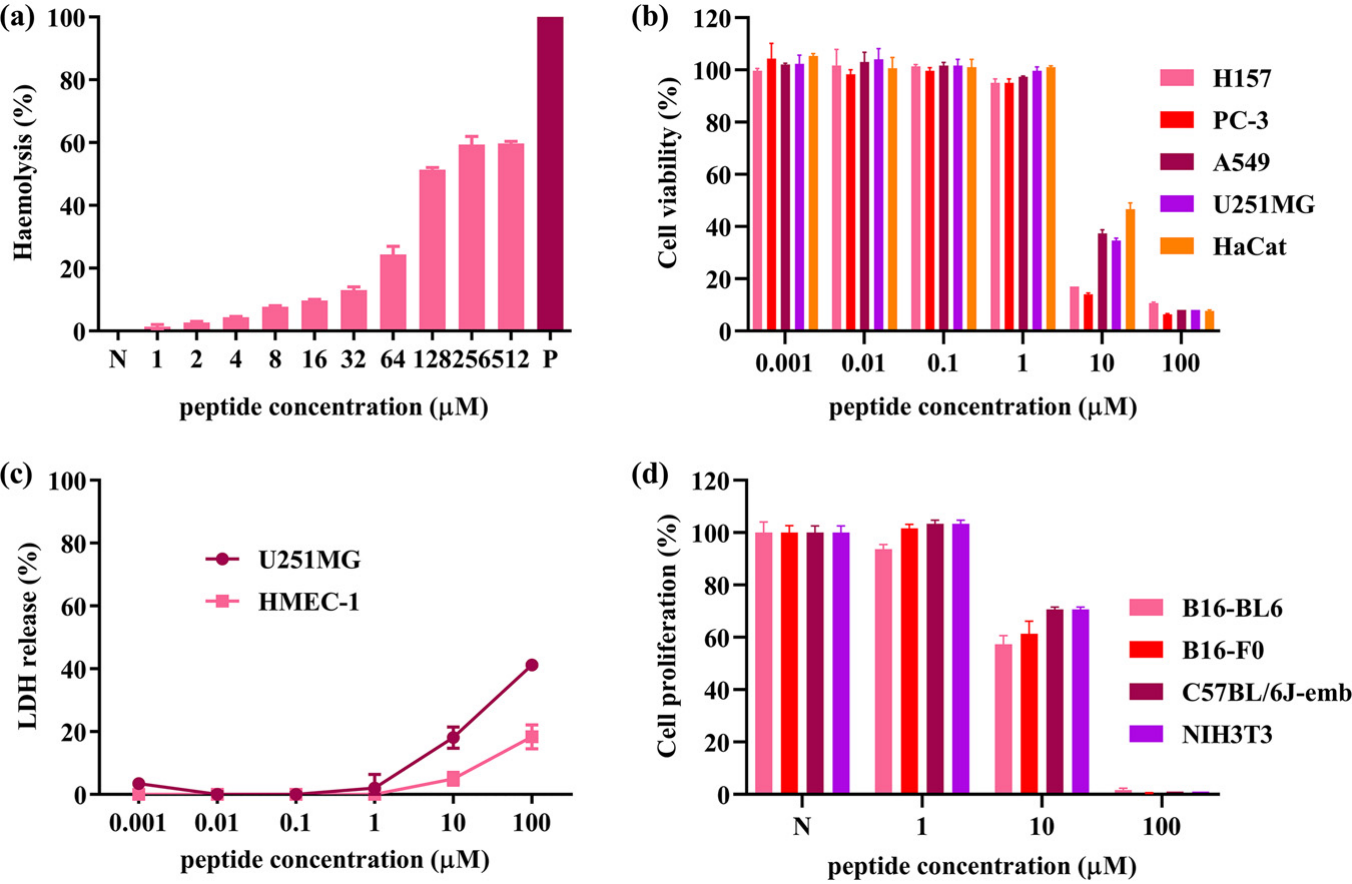
Hemolysis and cytotoxicity of Dermaseptin-AC. (a) Hemolysis of Dermaseptin-AC against horse erythrocytes. Hemolysis was calculated based on the measured OD value. (b) Cell viability under Dermaseptin-AC treatment (MTT assay). Cells were cultured with peptide at 37°C for 24 h and then incubated with MTT at 37°C for 4 h. Cell viability was calculated from measured OD values and untreated cells were set as 100% viable. (c) Cytotoxicity of Dermaseptin-AC against HMEC-1 and U251MG cells (LDH assay). Cells were incubated with peptide at 37°C for 24 h, and LDH release was calculated from the measured OD value. (d) Cell proliferation under Dermaseptin‐AC treatment. Cells were incubated with peptide at 37°C for 24 h and then enumerated. P represents positive control (1% Triton X-100) and N represents negative control (PBS). All data were analyzed by GraphPad Prism (Version 8.0.2), shown as mean ± SEM of 3 individual experiments with 3 replicates in each.

At concentrations of 2 μM, 4 μM, and 8 μM, the hemolysis rates of Dermaseptin-AC to horse red blood cells were 2.73%, 4.31%, and 7.47%. The HC_50_ (50% maximum hemolysis concentration) of Dermaseptin-AC against horse erythrocytes was 76.55 μM. The results of the 3-(4,5-dimethyl-2-thiazolyl)-2,5-diphenyl-2H-tetrazolium bromide (MTT) and cell proliferation assays showed that Dermaseptin-AC decreased cell viability and proliferation over 24 h, and had a slightly stronger effect on cancer cells than on normal cells. The cytoplasm of living cells contains lactate dehydrogenase (LDH), which cannot penetrate the cell membrane. Therefore, the integrity of the plasma membrane can be measured by LDH release. The results of LDH assay showed that Dermaseptin-AC could induce more LDH release from human glioblastoma cells U251MG (18.09% at 10 μM and 41.23% at 100 μM) than from human dermal micro vascular endothelial cell HMEC-1 (4.99% at 10 μM and 18.33% at 100 μM). The above data are shown in Fig. 5 and calculated IC_50_ values are summarized in Table 2.

**TABLE 2:** IC_50_ values of Dermaseptin-AC against mammalian (human and mouse) cell lines

Cell line	IC_50_ (μM, μg/mL)	Cell type
Human^*a*^		
HaCaT	8.43, 21.59	Keratinocytes
A549	6.21, 15.90	Non-small cell lung cancer
U251MG	6.14, 15.72	Glioblastoma
PC-3	3.39, 8.68	Prostate cancer
H157	3.22, 8.25	Non-small cell lung carcinoma
		
Mouse^*b*^		
NIH/3T3	∼18.26, ∼46.76	Embryo fibroblast
C57BL/6J-emb	∼18.26, ∼46.76	Embryonic
B16-F0	∼14.04, ∼35.96	Melanoma
B16-BL6	∼14.25, ∼36.49	Metastatic melanoma

a MTT assay.

b Proliferation assay.

### *In vivo* anti-MRSA activity.

**FIG 6:**
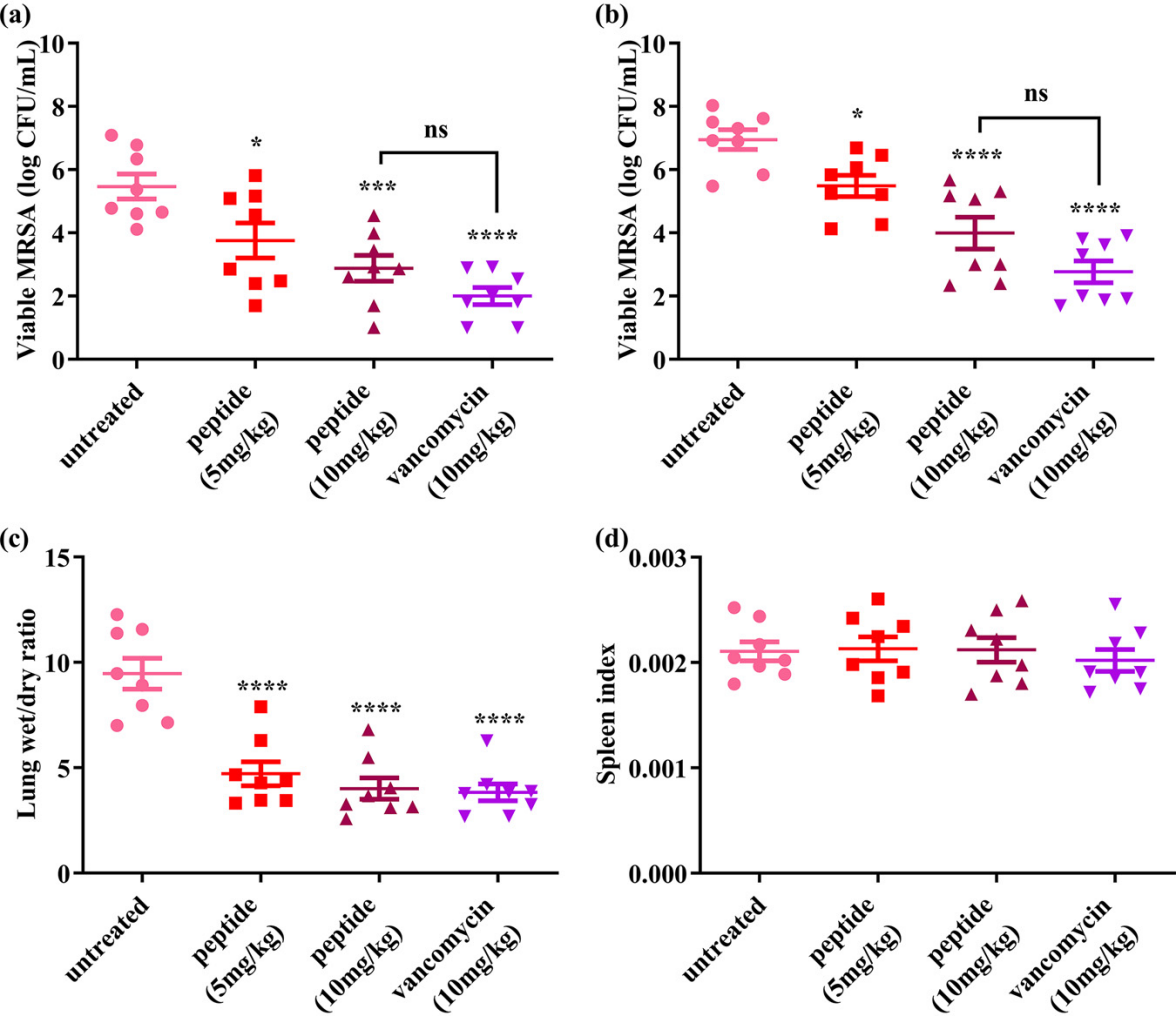
*In vivo* anti-MRSA activity of Dermaseptin-AC. Mice were nasally infected with MRSA (1 × 10^9^ CFU/mL, 20 μl) and treated by intraperitoneal injection after 24 h. Mice were euthanized after 24 h treatment. Viable MRSA in the BALF (a) and lung homogenate (b) of mice were counted. The left upper lobes of mice were weighed immediately and then dried at 60°C for 72 h. (c) Lung wet/dry ratio (ratio of fresh lung weight to dried lung weight) was calculated to estimate the degree of pulmonary edema. (d) Spleen index (fresh spleen weight/body weight) was calculated to estimate the immune status. All data were analyzed by GraphPad Prism (Version 8.0.2), shown as mean ± SEM with 8 replicates. Significant differences are indicated with asterisks (*, *P* < 0.05; ***, *P* < 0.001; ****, *P* < 0.0001). “ns” indicates no significance (*P* > 0.05).

MRSA was inoculated into the lungs of immunosuppressed mice, and drugs were administered by intraperitoneal injection. Results are shown in Fig. 6. Compared with those of the untreated group, the CFU burdens in bronchoalveolar fluid (BALF) and lung for each treated group were significantly reduced. Dermaseptin-AC was more active at 10 mg/kg than at 5 mg/kg (*P* = 0.0251 and *P* = 0.0005 for BALF; *P* = 0.0065 and *P* = 0.0002 for lung), and contributed a similar effect size to vancomycin at 10 mg/kg (*P* = 0.0922 for BALF and *P* = 0.0652 for lung). At the same time, the lung wet/dry ratios of all treated groups were significantly lower than those of the untreated group (*P* < 0.0001). There was no significant difference in the spleen index between untreated and treated groups.

### Intraperitoneal injection safety evaluation.

**FIG 7:**
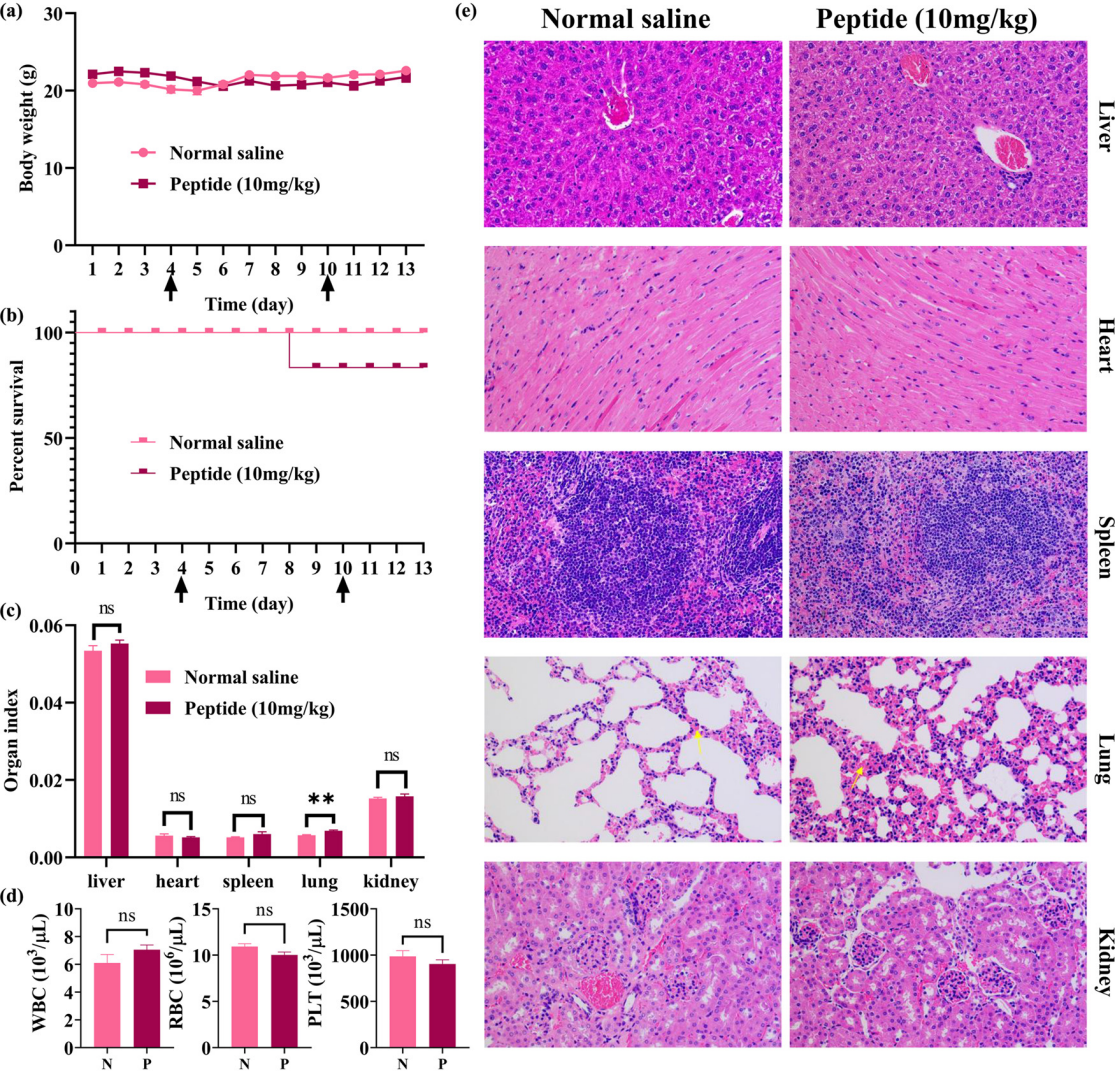
Intraperitoneal injection safety evaluation of Dermaseptin-AC. Mice were kept in SPF conditions for 3 days and then intraperitoneally injected with 10 mg/kg peptides or normal saline with equal volume for 7 days. Start and end days of the injection are indicated by arrows (**↑**). Mice were euthanized 3 days after final administration. Body weight (a) and % survival (b) were recorded every day. (c) The main organs were weighed immediately and organ index (ratio of fresh organ weight to body weight) was calculated (normal saline *n* = 5, peptide *n* = 4). (d) Blood cells, including white blood cells (WBC), red blood cells (RBC), and platelets (PLT) in mouse whole blood were counted (*n* = 4). (e) Photos (200×) of main organs tissues stained with hematoxylin and eosin were taken. Lung congestion is indicated by a yellow arrow. N represents normal saline and P represents peptide (10 mg/kg body weight). All data were analyzed by GraphPad Prism (Version 8.0.2), shown as mean ± SEM. Significant differences are indicated with asterisks (**, *P* < 0.01). “ns” indicates no significance (*P* > 0.05).

In the short-term *in vivo* safety experiment, healthy mice were intraperitoneally injected with either peptide solution (10 mg/kg) or normal saline for 7 days. One of the five mice in peptide group died on the fifth day of injection. The autopsy revealed that the cause of death was peritonitis, which may have been due to organ damage during the injection. After the injection was terminated, the mice were observed for another 3 days, and there was no abnormal behavior. The body weight of the mice in peptide group decreased compared to that of the mice in normal saline group. One blood sample in the saline group was hemolyzed due to improper operation in blood collection, so four samples in each group were finally analyzed. The results showed that the total numbers of red blood cells (RBC), white blood cells (WBC), and platelets (PLT) were not significantly different between the two groups (*P* = 0.0841, 0.3808, 0.3156). It was found that the lung index (ratio of fresh lung weight to body weight) of the peptide group was significantly increased compared with that of the normal saline group (*P* = 0.0022). Through hematoxylin-eosin staining, slight congestion was induced in the lung tissues of mice in peptide group. There were no significant differences in the indexes of other major organs. Results are shown in Fig. 7.

### Skin wounds safety evaluation.

**FIG 8:**
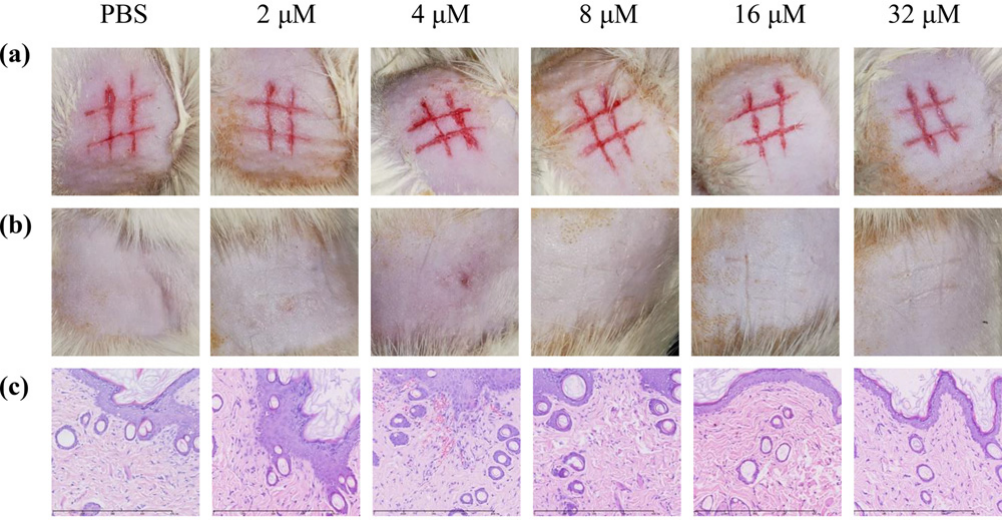
Skin wound safety evaluation of Dermaseptin-AC. The rat was kept in an SPF environment for 3 days and then de-haired with pet depilatory cream. Twenty-four hours after hair removal, the back skin of the rat was carefully scratched and six “#”-shaped wounds were made. After 24 h, a volume of 0.5 mL peptide solution of different concentrations and PBS was applied evenly. The treatment was then carried out every 24 h for a total of four times. The rat was euthanized on the fifth day after wound establishment and skin tissues were collected. Photos of newly established wounds (a), treated wounds (b), and tissues stained with hematoxylin and eosin (200×) (c).

Peptide solutions of different concentrations were applied to back skin wounds on a rat. The behavior of the rat was observed during treatment, and it did not appear to feel pain or irritation. There was also no scaly edema or erythema on the skin. Hematoxylin-eosin staining showed increased skin fibroblasts and new blood vessels in the wound area, with no inflammation, indicating that the skin healed well. Photos are shown in Fig. 8.

## DISCUSSION

In this study, a new AMP named Dermaseptin-AC was discovered from the skin secretion of *A. callidryas*. The predicted secondary structure indicated an amphipathic α-helix configuration of Dermaseptin-AC, and CD spectrum confirmed this prediction. In order to evaluate the possible use of Dermaseptin-AC against drug-resistant bacteria, the antibacterial activity and safety of Dermaseptin-AC were studied *in vivo* and *in vitro*.

Both the MICs and the MBCs of Dermaseptin-AC against S. aureus, E. faecalis, MRSA, E. coli, C. albicans were 2 μM. However, for P. aeruginosa and *K. pneumonia*, these values were higher (P. aeruginosa MIC = 4 μM, MBC = 8 μM; K. pneumoniae MIC = 2 μM, MBC = 8 μM). A possible reason is that P. aeruginosa and K. pneumoniae tend to secrete extracellular polysaccharide matrix, which hinders the effect of peptides on bacteria (16, 17). Studies have shown that capsular polysaccharides released by K. pneumoniae and alginate secreted by P. aeruginosa shield AMPs, causing conformational changes in them and preventing their interaction with microbial membranes (18). The results of SYTOX Green assay showed that Dermaseptin-AC increased the cell membrane permeability of MRSA at 2×MIC. However, for P. aeruginosa and K. pneumoniae, that concentration was 4×MIC. The biofilm of MRSA is also composed of extracellular polymers including proteins and polysaccharides (19). The results of MRSA biofilm-staining showed that Dermaseptin-AC inhibits the formation of MRSA biofilm from 4 μM, but eradicates mature biofilm from 256 μM. It can be inferred that biofilms can adsorb peptides and prevent them from reaching the bacteria, and that a higher concentration of peptides is required to reach the critical biofilm breaking point. Basically, Dermaseptin-AC showed very low membrane permeability under the MICs on all four strains tested. Based on this, it is speculated that the antimicrobial mechanism of Dermaseptin-AC on bacteria may include interfering with the membrane at low concentrations and destroying the membrane at high concentrations. This can be explained by the carpet model and the SMART model in the mechanisms of AMPs (20, 21). Interestingly, even at 4×MIC, Dermaseptin-AC did not induce high membrane permeability (only 44.6% ∼ 79.4%). It has been reported that the antibacterial activity of some AMPs depends not only on their effects on membranes, but also on some intracellular mechanisms (22). It is reasonable to speculate that Dermaseptin-AC has antibacterial mechanisms other than those that affect the membrane; this requires further verification.

Compared with the concentration of antibacterial activity, Dermaseptin-AC showed moderate hemolytic activity, because the HC_50_ was about 38 ∼ 19 times higher than the MICs. Furthermore, the antibacterial activity of Dermaseptin-AC against S. aureus and E. coli did not change in Mueller-Hinton broth (MHB) containing 5% serum. Therefore, it is speculated that Dermaseptin-AC could exert antibacterial effects through injection. In addition, whether in MTT assay, LDH release assay, or cell proliferation assay, Dermaseptin-AC was more cytotoxic to cancer cells than to normal cells. It is well known that lipid bilayers in bacterial membranes are mainly composed of lipids with negatively charged head groups, while mammalian cell membranes are mainly composed of zwitterionic phospholipids (23). It has also been proven that the plasma membrane lipids of cancer cells have increased anionic components compared to normal cells (24). The electrostatic attraction between the cationic peptide and the anionic component of the cell membrane is considered to be the most important factor in the selective function of AMPs (25). For Dermaseptin-AC, the four lysine residues in the peptide chain could act as positive charge donors. At the same time, the glycine in the peptide precursor could contribute to the formation of C-terminal amidation, which also enhances the electrostatic interaction (26, 27). The appropriate selectivity of AMPs is also related to their hydrophobicity, with strong hydrophobicity (i.e., exceeding the critical point) leading to a decrease in selectivity, thereby triggering hemolysis (28). Thus, the amino acid composition of Dermaseptin-AC gives it an appropriate selectivity, offering an available safety margin.

Our laboratory also found another AMP from *A. callidryas* skin secretions, which was named Dermaseptin-AC4 (SLWGKLKEMAAAAGKAALNAVNGLVNQ-NH_2_). Two synthetic peptides were designated as Dermaseptin-AC4a (SLWGKLKEMLAKAGKAVANAVNGLANQ-NH_2_) and Dermaseptin-AC4b (SLWGKLKEMLAAAGKAVANAVNGLANQ-NH_2_), based on Dermaseptin-AC4 (29). The *in vitro* antibacterial activity of Dermaseptin-AC4 and its analogues were much weaker than that of Dermaseptin-AC, possibly because Dermaseptin-AC is more hydrophobic, more positively charged, and more likely to form an α-helical structure when it contacts the cell membrane. At the same time, Dermaseptin-AC4 and its analogues were less hemolytic than Dermaseptin-AC. Though the MIC and MBIC of Dermaseptin-AC4a were higher than those of Dermaseptin-AC, the MBEC (minimum biofilm eradication concentration) of Dermaseptin-AC4a was lower. In the case where the positive charge, α-helix, and hydrophobicity were all weaker than those of Dermaseptin-AC, it seems that continuous hydrophobic moment of Dermaseptin-AC4a strengthened the biofilm eradicating effect. The structure and activity comparison of the above four peptides are shown in Fig. S1 and Table S1.

*In vitro* experiments indicate that Dermaseptin-AC has the potential for further application *in vivo*. Therefore, the *in vivo* antibacterial experiment was performed on mice with MRSA pneumonia. The results showed that Dermaseptin-AC could significantly reduce the pulmonary edema caused by infection as well as reducing viable bacteria burdens. Moreover, the *in vivo* efficacy of Dermaseptin-AC was close to that of vancomycin at same dose, indicating that it has the potential to become a new anti-MRSA drug. At the same time, the possible toxicity of Dermaseptin-AC was evaluated *in vivo*. Blood cell analysis results indicated that Dermaseptin-AC did not cause hemolysis in the mice. However, the increased lung index and slight lung tissue congestion suggest that lung damage might have been induced. Therefore, it is speculated that the abundant capillaries and sufficient blood flow in the lungs facilitate the antibacterial effect of Dermaseptin-AC, but that the lungs are also vulnerable to the toxic side effects.

Considering that Dermaseptin-AC might cause lung injury through systemic administration, topical medication might be a better choice, so the skin safety of Dermaseptin-AC was studied. No wound irritation was observed during 5-day topical application. The skin hematoxylin-eosin staining results also indicated good skin healing. Therefore, it is still promising to use Dermaseptin-AC for local treatment. Infection is one of the important factors which make chronic wounds difficult to heal, and long-term repeated infections and treatment accelerate the emergence of AMR (30). Hydrogel could enhance the stability of AMPs and prolong the contact time in the wound area (31, 32). The antibacterial activity of some biopolymers, such as chitosan, can act synergistically with AMPs (33). Thus, advanced hydrogel dressings that deliver AMPs are promising against drug-resistant infections in chronic wounds (34).

This research, however, is subject to several limitations. First, although seven microorganisms were involved in the *in vitro* antibacterial activity assay, only one strain of each microorganism was used instead of more clinically isolated strains. Therefore, in order to further clarify the possibility of clinical application of Dermaseptin-AC, more clinical isolates should be introduced in future study. Second, in the *in vivo* anti-MRSA experiment, the baseline of bacterial load after inoculation was not established. Therefore, the bacterial load of the treated group was only compared with that of the untreated group, which might be subject to bias in the 24-h growth of MRSA in mice. When investigating the possibility of future applications of Dermaseptin-AC in chronic wound infections, a baseline could be established through bacteria counting in wound exudate. Third, this study focused on bioactivities rather than on the antibacterial mechanisms of Dermaseptin-AC. Based on the data in this article, the intracellular mechanism should be studied in the future, and the influence of Dermaseptin-AC on the bacterial cell membrane should be observed.

In conclusion, a new AMP named Dermaseptin-AC was discovered from the skin secretion of *A. callidryas*. Dermaseptin-AC had effective broad-spectrum antibacterial activity and inhibited the formation of MRSA biofilm, showing appropriate selectivity to microorganisms and cancer cells *in vitro*. *In vivo* studies showed that Dermaseptin-AC had similar efficacy to that of vancomycin in immunosuppressed mice with MRSA pneumonia, but continuous injection might cause mild pulmonary congestion. It might be a better choice to deliver Dermaseptin-AC to chronic wound infections because it was not irritating to skin wounds.

## MATERIALS AND METHODS

### Skin secretion acquisition.

Specimens of adult *A. callidryas* were purchased from commercial sources and kept in amphibian facilities. According to the method of Tyler et al.(35), granular glandular secretions were collected from the dorsal skin by gentle transcutaneous electrical stimulation (5 V DC, 3 ms pulse width, 50 Hz) for 30 s. The secretions were then lyophilized by a Hetosicc 2.5 freeze drier (Heto, UK) and stored at −20°C. Sampling of skin secretions was carried out by Mei Zhou in accordance with the UK Animal (Scientific Procedures) Act of 1986. The project license was PPL2694 (issued by the Northern Ireland Department of Health, Social Services and Public Safety). This procedure has been reviewed by IACUC at Queen’s University Belfast and was approved on 1 March 2011.

### “Shotgun” cloning.

With the Dynabeads mRNA DIRECT kit (Biotech, UK), mRNA was isolated using 5 mg of freeze-dried skin secretions. A BD SMART RACE cDNA Amplification Kit (BD Clontech, UK) was used to generate full-length cDNA through reverse transcription reaction. The resultant cDNA was subjected to 3′-RACE PCR using a nested universal primer (5′-AAGCAGTGGTATCAACGCAGAGT-3′) and a sense primer (S1: 5′-GGCTTYCCTGAAGAAATCTC-3′) with same program as before (36). The PCR products were purified using a Cycle Pure Kit (Omega Bio-Tek, USA) and then cloned with pGEM Easy Vector System II (Promega, USA). Products were purified and expanded to prepare for the sequencing reaction, which was completed using BigDyeTerminator v 3.1 Cycle Sequencing Kit (Applied Biosystems, USA). The sequencing results were analyzed by Chromas software (version 2.6.4) and converted into amino acid sequences by the Expert Protein Analysis System (ExPASy). The open reading frame of peptide was compared with the NCBI-BLAST platform database (https://blast.ncbi.nlm.nih.gov/Blast.cgi), and multiple sequence alignments of similar peptide regions were performed through Clustal Omega (https://www.ebi.ac.uk/Tools/msa/clustalo/).

### Peptide synthesis and purification.

The peptide was synthesized by the fluorenylmethoxycarbonyl protecting group (Fmoc) solid-phase peptide synthesis method, using an automatic solid-phase synthesizer Tribute PS4 (Protein Technologies, USA). The synthetic peptide was lyophilized into powder and stored at −20°C. A sample solution (1 mg/mL) was prepared in a mixed solution of half A (80% acetonitrile (ACN), 19.95% water, 0.05% triflouroacetic acid [TFA]) and half B (0.05% TFA, 99.95% water), and then injected into a 1 cm × 25 cm Jupiter semipreparative C-18 column (Phenomenex, UK). The synthesized peptide with impurities was separated into different peaks by Cecil CE 4200 Adept (Cambridge, UK) gradient RP-HPLC (Waters 2489 UV/Visible detector, Waters 1525 Binary HPLC pump) using linear gradient elution. The linear gradient elution was from 100% A (0% B) to 0% A (100% B) at a flow rate of 1 mL/min over 80 min. The eluents of all peaks were collected separately, and each peak was verified by a MALDI-TOF mass spectrometer (Voyager, USA), using α-cyano-4-hydroxycinnamic acid (CHCA) as a matrix. The peak representing the target peptide was collected from the eluent of RP-HPLC and lyophilized as purified peptide.

### Secondary structure prediction and determination.

The secondary structure of Dermaseptin-AC was predicted by I-TASSER (37) and HeliQuest (38), and then determined by circular dichroism spectroscopy. Peptide was dissolved in 10 mM NH_4_Ac and 50% (vol/vol) trifluoroethanol (TFE)‐10 mM NH_4_Ac to reach a final concentration of 100 μM. A JASCO J-815 circular dichroism spectrometer (Jasco, UK) was used for detection (wavelength 190 ∼ 260 nm, scanning speed 200 nm/min, bandwidth 1 nm, data pith 0.5 nm).

### *In vitro* antimicrobial assays.

The *in vitro* antimicrobial activities were evaluated by agar and broth dilution methods (39). The MIC refers to the minimum concentration that can completely inhibit the growth of microorganism. The minimum bactericidal concentration (MBC) refers to the minimum concentration of the drug required to kill tested microorganism. Briefly, bacteria were inoculated in Mueller-Hinton broth (MHB; Solarbio, PRC) and then subcultured until reaching the logarithmic growth phase. Bacteria (5 × 10^5^ CFU/mL) were incubated with peptide, of final concentrations ranging from 64 μM to 1 μM, at 37°C for 18 ∼ 20 h. MBC was tested by subculturing 10 μl of clear incubated suspension on Mueller-Hinton agar (MHA; Solarbio, PRC) for 18 ∼ 20 h. S. aureus (NCTC 10788), E. coli (NCTC 10418), C. albicans (NCYC 1467), MRSA (ATCC 43300), E. faecalis (NCTC 12697), P. aeruginosa (ATCC 27853), and K. pneumoniae (ATCC 43816) were involved in this assay. The antibacterial activity of Dermaseptin-AC against S. aureus and E. coli was also tested in MHB containing 5% fetal bovine serum (FBS; Biological Industries, PRC).

### SYTOX Green staining assay.

MRSA, E. faecalis, P. aeruginosa, and K. pneumoniae were inoculated in MHB and incubated at 37°C overnight, and then subcultured in tryptic soy broth (TSB; Solarbio, PRC) until reaching the logarithmic growth phase by measuring optical density (OD) of the cultures at a wavelength of 550 nm. The bacterial suspension (1 × 10^8^ CFU/mL) was prepared with 5% TSB (dissolved in 0.85% NaCl water solution). The bacteria was permeabilized with 70% isopropanol and then resuspended in 5% TSB as a positive control. Peptide was diluted with 5% TSB for final concentrations of 1×MIC, 2×MIC, and 4×MIC. Bacterial suspension and peptide solutions or 5% TSB (negative control) were added to each well. The additional control consisted of 5% TSB without bacteria. The plate was incubated at 37°C in the dark for 2 h. After incubation, SYTOX Green nucleic acid stain (Molecular Probes, USA) was added to each well in darkness. After 5 ∼ 10 min of shaking, the plate was read with excitation at 485 nm and emission at 528 nm to obtain the fluorescent intensity (FI). Permeability was calculated using the following formula:

$$\text{Permeability}=\frac{\left(\text{FI sample }-\text{ FI additional control}\right)\;-\;(\text{FI negative control }-\text{ FI additional control})}{\left(\text{FI positive control }-\text{ FI additional control})\;-(\text{FI negative control }-\text{ FI additional control}\right)}\times 100\%$$

### Biofilm assay.

Crystal violet staining method was used to test the effect of Dermaseptin-AC on MRSA biofilm. MRSA was inoculated in TSB (Solarbio, PRC) and incubated at 37°C overnight, and then subcultured in TSB until reaching the logarithmic growth phase. The minimum biofilm inhibition concentration (MBIC) refers to the minimum concentration that can completely inhibit the formation of microbial biofilms. For the MBIC assay, peptide and MRSA suspension (5 × 10^5^ CFU/mL) were incubated together at 37°C for 24 h. The plate was rinsed twice and then stained with 0.1% crystal violet solution (Sigma-Aldrich, UK) for 15 min. The minimum biofilm eradication concentration (MBEC) refers to the minimum concentration of the drug required to remove the mature biofilm that has formed. For the MBEC assay, mature biofilms were pre-cultured, and then peptide was added to observe the eradication effect on the mature biofilms. MRSA suspensions (5 × 10^5^ CFU/mL) was added to a 96-well plate, and incubated at 37°C for 48 h to obtain mature biofilms, which were then rinsed twice by PBS. Mature biofilms were treated with peptide TSB solution at 37°C for 24 h. The plate was then rinsed and stained as in the MBIC assay. After air-drying overnight, stains were dissolved in 30% acetic acid (Sigma-Aldrich, UK) and the OD value of each well was recorded at 550 nm.

### Hemolysis assay.

Fresh horse blood (supplied by TCS Biosciences Ltd., UK) was centrifuged at 930 × *g* for 5 min in an Eppendorf Centrifuge 5430 (Eppendorf, Germany). The supernatant was removed, and the horse blood cells were resuspended with PBS. This process was repeated until the blood was clear, and finally a 4% erythrocyte suspension was prepared. The 2% Triton X-100 (Sigma-Aldrich, USA) solution was prepared as the positive control (final concentration was 1%). Erythrocyte suspension was mixed with either 2% Triton X-100 solution or with peptide solution of each concentration. All of the tubes were incubated at 37°C for 2 h and then centrifuged at 930 × *g* for 5 min. The supernatant of each tube was gently transferred into a 96-well plate, and OD value was recorded at 450 nm. The HC_50_ of peptide against horse erythrocytes were obtained through the GraphPad Prism (Version 8.0.2) calculation. Hemolysis was calculated using the following formula:

$$\text{Hemolysis}=\frac{\text{OD sample }-\text{ OD negative}}{\text{OD positive }-\text{ OD negative}}\times 100\%$$

### MTT assay and LDH assay.

Human non-small cell lung carcinoma cells (H157), human prostate cancer cells (PC-3), human glioblastoma cells (U251MG), human non-small cell lung cancer cells (A549), and human keratinocytes cells (HaCaT) were cultured in RPMI 1640 medium (Life Technology, UK) supplemented with 10% FBS (Sigma-Aldrich, USA) and 1% penicillin-streptomycin (100 unit/mL, 100 μg/mL; Sigma-Aldrich, USA) at 37°C and 5% CO_2_. Cells were seeded in a 96-well plate (5 × 10^3^ cells/well) and cultured for 24 h, then cultured in serum-free medium for 6 h. Peptide solutions with concentrations ranging from 100 μM to 0.001 μM were prepared in serum-free medium and incubated with the cells for 24 h. MTT 5 mg/mL solution (Thermo Fisher Scientific, USA) was added to each well and incubated for 4 h, after which the supernatant was removed from the wells and 100 μl dimethyl sulfoxide (DMSO) was added to each well. The plate was gently shaken until the crystals dissolved; OD value was measured at 570 nm. U251MG and human dermal micro vascular endothelial cells (HMEC-1) were cultured and treated with peptide under the same conditions. The lactate dehydrogenase (LDH) release was measured with a Pierce LDH Cytotoxicity Assay Kit (Thermo Scientific, UK).

### Cell proliferation assay.

Mouse embryo fibroblast cells (NIH/3T3), mouse embryonic cells (C57BL/6J-emb), mouse melanoma cells (B16-F0), and mouse metastatic melanoma cells (B16-BL6) were cultured in RPMI 1640 medium supplemented with 10% FBS and 1% penicillin-streptomycin (100 unit/mL, 100 μg/mL) at 37°C and 5% CO_2_. Cells were seeded in a 12-well plate (1 × 10^5^ cells/well) and cultured for 24 h. Peptide solutions with concentrations of 100 μM, 10 μM, and 1 μM were incubated with cells for 24 h. Cells were rinsed with PBS, collected by trypsin digestion, and then enumerated by Z1 Coulter Counter (Beckman Coulter, Japan).

### *In vivo* anti-MRSA experiment.

MRSA was cultured on blood agar at 37°C overnight. The following day, several typical colonies were subcultured to MHB 37°C overnight. BALB/c mice (8 weeks old, male) were purchased (SPF Biotechnology Ltd., PRC) and kept in special pathogen-free conditions. Animal care was performed in accordance with the guidelines of the Ministry of Science and Technology of China and the relevant ethical norms of Beijing University of Chinese Medicine (approval number: BUCM-4-2019080103-3101). The mice were injected intraperitoneally with 0.1 mL (0.5 mg) of cortisol (Bioruler, USA) in the morning and 0.2 mL (0.5 mg) of cyclophosphamide (Bioruler, USA) in the afternoon for 3 days to achieve an immunosuppressive state. Immunosuppressive mice were then instilled with MRSA (1 × 10^9^ CFU/mL, 20 μl) through the nose and randomly divided into four groups. One hour later, mice in each group were injected intraperitoneally with normal saline, 5 mg/kg peptide, 10 mg/kg peptide, and 10 mg/kg vancomycin (Bioruler, USA) dissolved in normal saline. Mice were euthanized after 24 h and their left lungs were ligated. A total volume of 1.2 mL of bronchoalveolar lavage fluid (BALF) was collected by infusing and withdrawing with cold PBS from cannulated mice trachea. The left lower lobe was homogenized with 0.5 mL cold PBS. The BALF and lung lobe homogenate were cultured on MHA, and colonies were counted to determine the bacterial burden. The left upper lobes of the mice were weighed immediately (wet weight) and then weighed after drying at 60°C for 72 h (dry weight). Lung wet/dry ratio (ratio of fresh lung lobes weight to dry lung lobes weight) was then calculated to estimate the status of pulmonary edema. The spleen was weighed immediately and the spleen index (ratio of fresh spleen weight to body weight) was calculated to estimate immune status. The above method refers to previously published work (40).

### Intraperitoneal injection safety experiment.

BALB/c mice (8 weeks old, male) were purchased (SPF Biotechnology Ltd., PRC) and kept in special pathogen-free conditions. Animal care was performed in accordance with the guidelines of the Ministry of Science and Technology of China and the relevant ethical norms of Beijing University of Chinese Medicine. After a 5-day adaptive maintenance, the body weights of mice were measured every day. On the fourth day, mice in each group were injected intraperitoneally with normal saline and 10 mg/kg peptide solution. The injection lasted for a week, and then the mice were kept under observation for 3 days. On the fourteenth day, the blood was obtained carefully through retro-orbital collection. Mice were then euthanized and main organs, including lung, heart, spleen, lung, and kidney were collected. Each organ was immediately weighed and the organ index (ratio of fresh organ weight to body weight) was calculated. The blood samples were analyzed by the MEK-7300P automated hematology analyzer (Nihon Kohden, Japan). The organ tissues were fixed in paraformaldehyde fixative (Servicebio, PRC) and then stained with hematoxylin and eosin.

### Skin wounds safety experiment.

An SD rat (8 weeks old, male) was purchased (SPF Biotechnology Ltd., PRC) and kept in special pathogen-free conditions. Animal care was performed in accordance with the guidelines of the Ministry of Science and Technology of China and the relevant ethical norms of Beijing University of Chinese Medicine. Six small “#"-shaped wounds were created by scratching the epidermal layer with a needle on the back skin of the rat. A volume of 5 mL of peptide solutions (2 μM, 4 μM, 8 μM, 16 μM, 32 μM) and PBS was applied to the wounds for 5 days. After the last administration, the rat was euthanized and skins of the wound area were collected for tissue fixation and hematoxylin-eosin staining.

### Statistical analysis.

Statistical analysis was performed using GraphPad Prism (Version 8.0.2). All results are presented as means ± standard errors of the mean (SEM) determined by one‐way ANOVA and unpaired *t* test. Significant differences are indicated with asterisks (*P < 0.05, **P < 0.01, ***P < 0.001, ****P < 0.0001).

### Data availability.

The GenBank accession number of Dermaseptin-AC is MN809621 (https://www.ncbi.nlm.nih.gov/genbank/). The raw data supporting the conclusions of this article will be made available by the authors, without undue reservation, to any qualified researcher.
