# Supplementary material for: In Vitro and In Vivo Studies on the Antibacterial Activity and Safety of a New Antimicrobial Peptide Dermaseptin-AC
Source: Microbiol Spectr. 2021 Dec 15;9(3):e01318-21. doi: 10.1128/Spectrum.01318-21 (PMC8672897; doi:10.1128/Spectrum.01318-21)
Supplement: SUPPLEMENTAL FILE 1 — Supplemental material. Download SPECTRUM01318-21_Supp_1_seq1.pdf, PDF file, 0.4 MB [file spectrum01318-21_supp_1_seq1.pdf]

Supplementary data

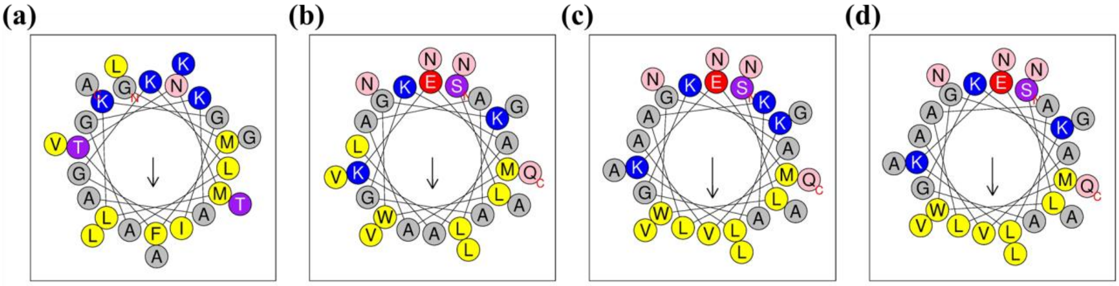

Figure S1. HeliQuest wheel of **(a)** Dermaseptin-AC, **(b)** Dermaseptin-AC4, **(c)** Dermaseptin-AC4a, **(d)** Dermaseptin-AC4b

Table S1. Structure and activity comparison of four peptides

AMPs	α-Helix (%)	Net charge	Hydrophobicity(H)	hydrophobic moment(μH)	MIC (μM)	MBC (μM)	MBIC (μM)	MBEC (μM)	HC_{10} (μM)
DRP-AC	50.2	5	0.445	0.378	2~4	2~8	4	256	8.5
DRP-AC4	22.4	3	0.341	0.404	8~64	8~128	32	>256	26.25
DRP-AC4a	26.9	4	0.293	0.526	8~32	8~128	16	64	14.49
DRP-AC4b	38.8	3	0.341	0.492	16~128	16~128	32	256	21.53

DRP indicates Dermaseptin
